# Lessons learned about the effective operationalization of champions as an implementation strategy: results from a qualitative process evaluation of a pragmatic trial

**DOI:** 10.1186/s13012-020-01048-1

**Published:** 2020-10-01

**Authors:** Arwen E. Bunce, Inga Gruß, James V. Davis, Stuart Cowburn, Deborah Cohen, Jee Oakley, Rachel Gold

**Affiliations:** 1 OCHIN, Inc., 1881 SW Naito Pkwy, Portland, OR 97201 USA; 2 Kaiser Permanente Center for Health Research, 3800 N Interstate Ave, Portland, OR 97227 USA; 3 School of Medicine, Oregon Health and Science University, 3181 SW Sam Jackson Park Rd, Portland, OR 97239-3098 USA

**Keywords:** Implementation strategies, Practice change, Clinical champions, Reflexivity

## Abstract

**Background:**

Though the knowledge base on implementation strategies is growing, much remains unknown about how to most effectively operationalize these strategies in diverse contexts. For example, while evidence shows that champions can effectively support implementation efforts in some circumstances, little has been reported on how to operationalize this role optimally in different settings, or on the specific pathways through which champions enact change.

**Methods:**

This is a secondary analysis of data from a pragmatic trial comparing implementation strategies supporting the adoption of guideline-concordant cardioprotective prescribing in community health centers in the USA. Quantitative data came from the community health centers’ shared electronic health record; qualitative data sources included community health center staff interviews over 3 years. Using a convergent mixed-methods design, data were collected concurrently and merged for interpretation to identify factors associated with improved outcomes. Qualitative analysis was guided by the constant comparative method. As results from the quantitative and initial qualitative analyses indicated the essential role that champions played in promoting guideline-concordant prescribing, we conducted multiple immersion-crystallization cycles to better understand this finding.

**Results:**

Five community health centers demonstrated statistically significant increases in guideline-concordant cardioprotective prescribing. A combination of factors appeared key to their successful practice change: (1) A clinician champion who demonstrated a sustained commitment to implementation activities and exhibited engagement, influence, credibility, and capacity; and (2) organizational support for the intervention. In contrast, the seven community health centers that did not show improved outcomes lacked a champion with the necessary characteristics, and/or organizational support. Case studies illustrate the diverse, context-specific pathways that enabled or prevented study implementers from advancing practice change.

**Conclusion:**

This analysis confirms the important role of champions in implementation efforts and offers insight into the context-specific mechanisms through which champions enact practice change. The results also highlight the potential impact of misaligned implementation support and key modifiable barriers and facilitators on implementation outcomes. Here, unexamined assumptions and a lack of evidence-based guidance on how best to identify and prepare effective champions led to implementation support that failed to address important barriers to intervention success.

**Trial registration:**

ClinicalTrials.gov, NCT02325531. Registered 15 December 2014.

Contribution to the literature

- These findings show that detailed, contextualized reporting of how implementation strategies operate is critical to replicating successful implementation outcomes.
- The implementation science literature lacks guidance on how to operationalize the champion role; these findings advance our understanding of how to effectively do so.
- The findings suggest that reflexivity, or the active querying of assumptions affecting decisions during the design phase—particularly as these assumptions relate to likely barriers and facilitators (determinants) to intervention uptake—is essential to avoid a mismatch between how implementation strategies are operationalized and setting-specific determinants.

## Introduction/background

Implementation strategies are techniques designed to promote the uptake of clinical interventions into practice [1] by targeting key modifiable barriers and facilitators (called implementation determinants) to adoption of these interventions [2, 3]. Implementation science seeks to develop evidence to improve the accurate identification of (i) implementation barriers relevant to specific interventions [4–6], and (ii) implementation strategies likely to address such barriers [5, 7, 8]. While the evidence on effective implementation strategies is growing, much remains unknown about how best to select and enact these strategies for a given context. Currently, such planning usually focuses on which implementation strategies to use, typically informed by knowledge of/hypotheses about likely implementation barriers [1, 3, 9–15], existing evidence about the effectiveness of the strategies [1, 3, 9, 13–16], the experience of the implementation team [17], guidance from conceptual models [6, 18–25], and pragmatic concerns [1, 3, 9, 15] including available resources [4, 5, 13, 26]. However, these sources generally do not provide detailed guidance on how these strategies should be operationalized to maximize their impact.

The Study of Practices Enabling Implementation and Adaptation in the Safety Net (SPREAD-NET) involved a set of implementation strategies, selected based on evidence demonstrating their ability to support practice change, their potential scalability, and pragmatic considerations (e.g., cost). These strategies included (i) creating and distributing educational materials, (ii) conducting educational meetings and ongoing trainings, (iii) using train-the-trainer strategies, (iv) facilitation, and (v) identifying and preparing champions [27]. The study hypothesized that more intensive implementation support (e.g., more trainings; adding practice facilitation) would lead to greater improvements in the targeted outcomes. As such, the study was designed to test the impact of additive support, not the efficacy of any individual implementation strategy. The main SPREAD-NET analyses—of association between study outcomes and degree of implementation support—found that more intensive implementation support was not associated with greater improvements in the targeted outcomes (rates of guideline-concordant cardioprotective prescribing) [28].

For the manuscript presented here, we re-analyzed the study’s mixed-methods data to identify factors associated with differences in study outcomes in individual community health centers. As initial results indicated that the interplay between study implementers (clinic staff tasked with leading change processes) and organizational context was key to the success of a given community health center, we then explored the organization-specific pathways through which these implementers promoted practice change. We note that the use of champions [29–32] was one of the parent study’s implementation strategies chosen a priori; the intention was that the study implementers would act as champions. In this paper, however, and per implementation science definitions [18, 27, 31], we use the term “champion” to describe only those study implementers who demonstrated a sustained commitment to implementation activities. In other words, all clinic staff tasked with leading change processes were study implementers, but only some acted as champions.

Little has been reported on how to effectively prepare people for the champion role, or on the specific pathways through which champions enact change [31, 33, 34]. This paper presents a detailed assessment of how study implementers/champions impacted prescribing behavior in varied settings, to better understand how this role is optimally operationalized, and thus advance the specification and preparation of champions as an implementation strategy.

## Methods

### Study setting and design

The SPREAD-NET study compared the effectiveness of increasingly intensive implementation strategies at supporting community health centers’ adoption of a suite of clinical decision support tools called the cardiovascular disease (CVD) bundle. The CVD bundle included point-of-care alerts and panel management data tools promoting cardioprotective prescribing guidelines for patients with diabetes [28]. It was activated in the study clinics’ shared EHR in June 2015, and modified in May 2017, when CVD risk calculation [35] was added to the logic underlying its alerts.

Twenty-nine clinics managed by 12 community health centers in six states participated in the study (a single community health center may encompass multiple clinics). All study community health centers were members of OCHIN, Inc., a non-profit organization based in Portland, OR, that provides a shared Epic© EHR to > 600 US clinics. The community health centers were cluster-randomized into one of three arms; the arms received increasingly intensive implementation support designed to promote uptake of the CVD bundle (Table 1) (we recognize that clinical decision support can be considered an implementation strategy [27]; here, the cVD bundle is the innovation targeted by he strategies under comparison).

**Table 1:** Implementation support by study arm

Expert recommendations for implementing change (ERIC) implementation strategy	Arm 1	Arm 2	Arm 3
	Low-intensity support (***n*** = 9 clinics)	Medium-intensity support (***n*** = 11 clinics)	High-intensity support (***n*** = 9 clinics)
**Identify and prepare champions** - One or more study implementers to lead implementation activities and liaise with the study team	x	x	x
**Develop and distribute educational materials** - Implementation toolkit covering how to use CVD bundle components and tips on practice change	x	x	x
**Conduct ongoing trainings** - Annual webinars on the CVD bundle	x	x	x
**Conduct educational meetings/use train the trainer strategies** - 2-day, in-person training focused on the use of the CVD bundle and implementation toolkit		x	x
**Conduct ongoing trainings** - Quarterly webinars with content based on training needs		x	x
**Facilitation** - Ongoing practice facilitation from an implementation specialist			x

All study community health centers were required to identify a staff member to engage with the study team and lead the clinic’s efforts to support the use of the CVD bundle—a study implementer. Based on known attributes of effective champions [30, 31, 36–38], and our past experience [39], we suggested (but did not define or require) selecting implementers with involvement in and enthusiasm about quality improvement activities, credibility, and influence at the clinic, and interest in diabetes/cardiovascular care. One staff member could be the study implementer for up to three clinics in a single community health center. Each implementer was expected to participate in regular telephone check-ins with the study team, and those in arms 2 and 3 to attend the in-person training. Each community health center’s leadership chose study implementers based on these parameters and their own needs; some implementers volunteered for the role while others were assigned. Once selected, the research team explained the study implementers role to them as (i) acting as a resource/champion for any SPREAD-NET-related activities their clinic chose to implement (what/when/how up to each clinic), and (ii) acting as a liaison between the community health center and the study team. If the primary study implementer was not a clinician, the community health centers were asked to identify a clinician to serve as a role model/resource; that person was also invited to the in-person training. Of the 11 participating community health centers, five chose physicians as the primary study implementer. Of the remaining six community health centers, three chose physicians as the additional clinical implementation resource, one chose a nurse practitioner, and two opted not to fill the role.

### Data collection

The SPREAD-NET study followed a convergent mixed-methods design [40] in which quantitative and qualitative data were collected concurrently and merged for interpretation. As noted in the “Introduction/background” section, the study’s primary hypothesis—that implementation support provided with increasing intensity by study arm would be associated with additive improvements in the study outcomes—was not realized [28]. The analyses presented here used the same data sources as in the main study analysis to investigate factors associated with significantly improved outcomes in individual study community health centers (rather than across study arms). Data sources are summarized below.

#### Quantitative

Quantitative data were extracted from OCHIN’s EHR database. These data were used to measure the proportion of patients in a given community health center who had diabetes mellitus (DM), were indicated for a statin per care guidelines (denominator), and had a prescription for a statin (numerator), calculated monthly.

#### Qualitative

**Fig. 1:**
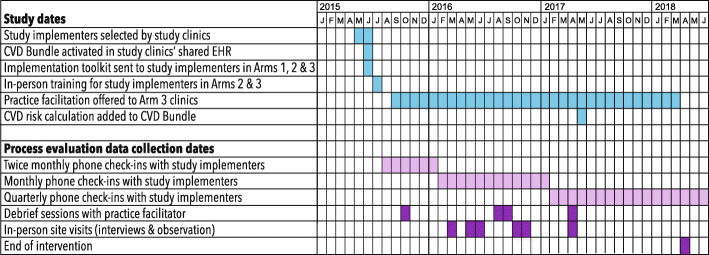
Study and data collection timeline

The 3-year qualitative process evaluation was conducted from August 2015, through July 2018. Figure 1 illustrates the intersection of the study timeline and process evaluation data collection points.

The evaluation design was informed by the practice change model [41], a conceptual model that seeks to identify the elements and interrelationships critical to practice change, and followed a grounded theory approach [42, 43] in which we attempted to understand the ongoing implementation experience from the perspective of study clinic staff. Our team’s qualitative researchers called the primary implementers twice a month in the 6 months after the start of the intervention in July 2015; conversations were loosely based on a question guide but were designed to be flexible and follow the implementers’ lead. We subsequently reduced call frequency to monthly, then quarterly, to reduce the participation burden. Four hundred and thirteen calls were conducted over the 3-year evaluation. We also conducted 2-day site visits with eight of the 12 community health centers, at 9 to 22 months post-intervention start. Sites were purposively chosen to maximize variation in implementation processes. During these visits, we interviewed clinic leadership, clinicians, and staff about individual- and clinic-level prescribing practices, use of the CVD bundle, barriers and facilitators to its adoption, and organizational approaches to practice standardization. The number of interviews per site ranged from 7 to 15, determined by clinic size and staff availability. We conducted three debrief sessions with the study practice facilitator (a member of the research team who provided ongoing practice facilitation to arm 3 clinics; Table 1) to learn about her experiences at the arm 3 clinics. Shortly after the intervention’s end, we conducted additional phone interviews with five clinic leaders to gain additional perspectives on the implementation process. All calls and interviews were recorded and professionally transcribed for analysis.

### Data analysis

The secondary analysis of the SPREAD-NET data presented here occurred in two rounds. We first used the coding structure created as part of the parent study to conduct a preliminary exploration of the qualitative data by community health center [40] to identify factors that distinguished between individual community health centers reporting more or less robust implementation activities. Robustness was characterized by the time and effort that a given organization put into implementation activities, and its willingness to adapt its approach as necessary; these factors were determined during the check-in calls with study implementers and interviews of clinic staff, and informed by on-site observation during site visits. Concurrently, quantitative analysis was performed to identify which community health centers (if any) demonstrated statistically significant improvements in guideline-concordant statin prescribing. Next, we compared those quantitative and qualitative results, then re-immersed ourselves in the qualitative data to better understand observed associations between improved prescribing and contextual/implementation variables. Details on the quantitative and qualitative analysis methods are provided below.

#### Quantitative

As with the main study analysis [28], we used a difference-in-difference (DiD) [44] approach to evaluate pre-/post-change in prescribing rate(s). For this analysis, we evaluated the change in statin prescribing rates by community health center (rather than study arm). All analyses reflect tests of statistical significance with a two-sided *α* of 0.05 and were conducted using SAS Enterprise Guide 7.15.

#### Qualitative

Coding was conducted iteratively throughout the parent study. A preliminary code list was created by the lead qualitative researcher (AB) 1 year into data collection. This draft list was reviewed and refined by the three-person qualitative team into a codebook; each team member then coded an identical set of transcripts and compared the results to identify items needing revision. After agreed-upon codes were applied consistently across all coders, independent coding assignments were made. The team double-coded five percent of the qualitative data as a quality check, resolving inconsistencies through team discussion and consensus and updating the codebook as necessary. Coding was conducted using the QSR NVivo software, guided by the constant comparative method [45, 46]. Coding was completed concurrently, but blinded to, quantitative analysis testing study hypotheses.

When the main study analyses found that increasingly intensive implementation support did not yield additive benefits [28], we designed and conducted the secondary analysis presented here to assess factors that might explain differences by community health center irrespective of amount of support received. We first identified factors potentially key to adoption success, based on knowledge of the clinics, data from two researchers involved in data collection throughout the study (AB, JD), and review of all qualitative data by a researcher who joined the study post-data collection (IG). Factors initially considered included community health center staff’s EHR proficiency/skills; community health center culture of quality improvement/practice change; competing quality improvement initiatives; organizational approach to standardization and emphasis on evidence-based care; staff/study implementer turnover; community health center-specific implementation approach; and study implementer characteristics and level of engagement. We then created community health center-specific reviews for each relevant code (e.g., implementation strategies and barriers; EHR optimization; point person; leadership and staff buy-in; standardization; practice change; context setting, culture, churn), and summarized this data by community health center. The 3-year data collection period allowed us to observe changes in implementation activities, contextual factors that impacted the implementation and implementer perspectives over time, all of which informed and enriched our understanding of the process of implementation at each site.

Based on this analysis, we identified a set of interconnected factors related to characteristics of the study implementer and the organizational context that differentiated those community health centers that did and did not demonstrate a significant increase in guideline-concordant statin prescribing in the quantitative results. We then conducted multiple cycles of immersion crystallization [47] in which qualitative team members read and re-read the transcripts and code reports, emerged to reflect on and discuss overarching themes and patterns, then delved back into the data to explore connections, confirm/disconfirm insights and interpretations, and flesh out our understanding of the essential attributes of study implementers and the mechanisms through which they successfully influenced prescribing practice at their organization.

The standards for reporting qualitative research (SRQR) was used to guide the reporting of qualitative findings (see Additional file) [48]. This study was approved by the Kaiser Permanente Northwest Institutional Review Board.

## Results

Five community health centers demonstrated statistically significant increases in guideline-concordant statin prescribing over the course of the study (Table 2).

**Table 2:** Results of adjusted difference-in-difference model for statin prescribing, by CHC and study arm

Study arm	CHC	Adjusted rate ratio	Lower 95% CI	Upper 95% CI
**1**	**#1**	**1.23**	**1.16**	**1.29**
1	#2	1.03	0.95	1.12
1	#3	1.14	0.99	1.32
1	#4	1.01	0.96	1.05
**2**	**#5**	**1.06**	**1.02**	**1.10**
**2**	**#6**	**1.22**	**1.14**	**1.30**
2	#7	0.90	0.80	1.02
**2**	**#8**	**1.18**	**1.08**	**1.29**
3	#9	1.10	0.99	1.22
3	#10	1.01	0.97	1.06
3	#11	0.99	0.92	1.06
**3**	**#12**	**1.09**	**1.02**	**1.16**

Results in bold indicate statistical significance at the *α* = 0.05 level

### The intersection of organizational investment and study implementer

Several factors, in combination, were associated with significant pre-post increases in guideline-concordant statin prescribing among the community health centers noted in Table 2. All were related to the characteristics of the study implementers and the organizational support they received. They included the following:

- *Engagement*: Interest in and willingness to promote the intervention
- *Influence*: Sufficient social capital to foster trust and the authority to prioritize implementation and stimulate practice change
- *Credibility*: Conferred through prescribing privileges
- *Capacity*: Time—and understanding of diabetes and cardiovascular care—sufficient to effectively advocate for the intervention

At each community health center that demonstrated a significant improvement in study outcomes, one or two implementers emerged as the de facto champions (see case studies, below). As noted in the “Introduction/background” section, we use the term champion to describe only those study implementers who demonstrated a sustained commitment to implementation activities [18, 27, 31] (Table 3). Study implementers who emerged as champions demonstrated each of the above elements (though at one community health center, the implementers were clinical pharmacists whose role included advising providers on prescribing decisions).

**Table 3:** Staff role of study implementers and emergent champions by CHC

CHC#	Appointed		Emergent champion
	Primary study implementer	Additional clinician implementer	
**1**	**Clinic 1: Clinic medical director/practicing physician**^**a**^ **Clinic 2: Successive RNs**	**N/A**	**Yes**
2	Practicing advanced practice provider, succeeded by clinic project coordinator	N/A	No
3	RN care manager^a^	Practicing physician	Yes
4	2 Clinical applications specialists, succeeded by clinical applications supervisor	CEO/advanced practice provider	No
**5**	**4 EHR technical support staff**	**Medical director/practicing physician**^**a**^	**Yes**
**6**	**Practicing physician**^**a**^	**N/A**	**Yes**
7	Practicing physician	N/A	No
**8**	**Successive advanced practice providers (2nd provider**^**a**^**)**	**Practicing physician**	**Yes**
9	Clinic 1: Practicing physician Clinic 2: RN	N/A	No
10	Clinic 1: Director of performance improvement and population health/RN^a^ Clinic 2: Support staff manager Clinic 3: Clinic manager Clinic 4: Clinic manager Later succeeded by physician (no longer practicing) for all clinics	Practicing physician	Yes
11	Clinical data analyst, succeeded by clinic manager, succeeded by MA supervisor	Chief medical officer, practicing physician	No
**12**	**Clinical pharmacists at their respective clinics**^**a**^	**Practicing advanced practice provider**	**Yes**

Bolded rows indicate statistical significance at the *α* = 0.05 level

^a^Denotes emergent champion

We defined organizational support as the creation of an environment within which implementation activities could be expected to be taken seriously by clinic staff. In many cases, this support was indicated by the selection of a staff member with the potential to be an effective champion (as described above; many of the necessary qualities were suggested by the study team in the initial study communications). Community health centers that demonstrated organizational support also promoted the use of the CVD Bundle and/or guideline-concordant statin prescribing. While organizational support, or the lack thereof, could take many forms—as illustrated below—implementation success depended on both the presence of champions with the aforementioned attributes and the implicit or explicit backing of clinic leadership, and the interaction of the two.

Community health centers that did not show a significant improvement in prescribing rates lacked either an emergent champion, and/or organizational support. Implementers at these community health centers often were less engaged with the study/CVD bundle; did not have adequate influence at their clinic to be effective change agents for this intervention; and/or did not have a clinical background (and therefore the credibility to affect provider care decisions). Two community health centers (see Table 3) did have champions that met each of the elements noted above apart from prescribing privileges (both were RNs), but implementation approaches and organizational priorities precluded their ability to effectively promote the targeted practice change.

While the combination of the above implementer characteristics—engagement, influence, credibility, and capacity—and organizational support appeared key to enabling practice change, each community health center—champion combination followed unique implementation paths suited to the particular context. Case studies of the seven community health centers with emergent champions, five of whom achieved significant improvements and two who did not, illuminate this diversity.

### Community health centers that significantly increased guideline-concordant statin prescribing for patients with diabetes

#### Community health center #1

Implementation efforts were led by a medical director [credibility], who had advocated for the organization’s participation in the study, and were effective primarily due to her efforts, position, and influence within the organization: she had a long history at the community health center and was respected and trusted by providers and staff [influence]. Despite practicing exclusively at the clinic where she served as medical director, she was instrumental in promoting the adoption of the targeted care guidelines at both clinics in the study. She was actively engaged in the implementation, conducting multiple trainings for providers and discussing the intervention, relevant guidelines, and the CVD bundle at organization-wide meetings [engagement, capacity]. She also led by example, consistently using the CVD bundle tools and developing relevant plan-do-study-act tests of change [engagement]. She worked closely with and directed the activity of an RN-turned EHR technical support staff member, who incorporated the CVD bundle into new staff trainings.

#### Community health center #5

Implementation efforts were directed by the organization’s medical director, who had made the decision to participate in the study, and supported by EHR technical staff at each clinic who were assigned to the implementer role by the medical director. This community health center achieved a significant increase in guideline-concordant statin prescribing through the sustained, active engagement of a leadership figure within an organizational structure that prioritized hierarchy and standardization [influence]. This medical director, a practicing physician [credibility], was actively involved in developing reports that identified patients in a given providers’ panel who were indicated for but not on the target medications. These reports were regularly distributed to providers, and the medical director met with providers individually to review them [engagement, capacity]. She also incorporated statin guideline prescribing performance into provider evaluations [engagement]. She standardized EHR screen configurations organization-wide to maximize viewing the point-of-care alerts, and had most non-CVD bundle alerts turned off to minimize alert fatigue [engagement]. She modified the statin dosing table provided in the SPREAD-NET toolkit to include locations where statins were available at low cost, and taped it to providers’ monitors for easy access [engagement, capacity]. Under her direction, links to a CVD risk calculator were added to the organization’s EHR before it became available as part of the CVD bundle [engagement]. She integrated explicit cardioprotective prescribing guidance into the community health center’s residents’ training [engagement, capacity]. Under her guidance and supported by the EHR technical support staff, each clinic conducted outreach to patients who were (over)due for diabetes care; the intensity of this effort varied by clinic and care team.

#### Community health center #6

Implementation efforts were enacted by an experienced, well-regarded physician [credibility] who had practiced at one of the clinics in this organization for many years and had previously served as medical director for the community health center [influence]. This provider was interested in research and volunteered to fill the study implementer role; her schedule already included 4 h a week of administrative time designated to community and research work [capacity]. This gave her the flexibility to innovate in her own practice and effect change through one-on-one engagement with other providers, including sharing alternative workflows for diabetes care, within an organizational culture that afforded substantial provider autonomy. In addition to experimenting with different workflows for diabetes care, this physician also met individually with all providers at both clinics, including during new provider onboarding, for a short, over-the-shoulder introduction of the study and the CVD bundle, and demonstration of the tools [engagement].

#### Community health center #8

Early implementation activities were limited to an introductory presentation to providers and occasional reminders during staff meetings. This community health center replaced their study implementer approximately 1 year into the study, shifting the role to a provider who was newer to the organization but brought a wealth of experience in diabetes care [credibility]; both providers were appointed to the implementer role by organizational leadership. The community health center’s increase in guideline-concordant statin prescribing appeared to be due to a combination of awareness-raising efforts by this clinician, coupled with an increasing organization-wide emphasis on standardization and quality improvement throughout the study period [influence]. During this time, the organization was working on becoming an accredited patient-centered medical home, with an attendant focus on practice change capacity. The new implementer brought renewed focus to the statin guidelines, often discussing prescribing recommendations at organization-wide provider meetings [engagement, capacity]. She also requested and received the addition of a link to a CVD risk calculator on staff computers, and notified providers when it was added to the CVD bundle [engagement, capacity].

#### Community health center #12

Implementation efforts were led by two clinical pharmacists, one of whom was the clinic’s director of clinical pharmacy. The pharmacy director chose to lead implementation activities at the main clinic; the second clinical pharmacist was asked to take on the role at the second clinic. Success at this site was largely due to the strength and credibility of the clinical pharmacy program at the organization, the dedicated time the pharmacists were able to spend supporting guideline-concordant statin prescribing [capacity], and parallel awareness-raising by clinical leaders that resulted in an increased emphasis on relevant prescribing guidelines by multiple influential actors at the organization. The clinical pharmacy department was well-resourced, trusted [influence], and guideline-focused. The clinical pharmacists incorporated updated statin guidelines into pharmacy protocols, met individually with patients to review their medications, reviewed patient charts for guideline-concordant prescribing [credibility], and followed up with providers in conversations or messages, as well as discussing the CVD bundle and relevant guidelines at clinic meetings [engagement]. The clinical pharmacists worked outside the daily patient encounter workflow, which challenged their ability to stimulate change at the provider and team level. However, the organization’s medical director and the chief operating officer, who had formerly worked as an RN at the organization, ensured that the statin guidelines were discussed and debated at provider meetings.

### Community health centers that did not significantly increase guideline-concordant statin prescribing for patients with diabetes

#### Community health center #10

Implementation efforts were coordinated by an RN who served as the director of quality improvement for the community health center; she volunteered for the role and acted as the primary study implementer for the largest of the four clinics taking part in the study. The remaining three study implementers, all of whom held patient-facing administrative roles, were asked to manage implementation activities at the other three clinics. The four study implementers initially met multiple times to plan their implementation approach, introduced the CVD bundle to care teams at all four clinics, and identified and worked with a single provider to initiate a pilot project using the CVD bundle within his own patient panel. These efforts, however, never gained momentum. Organizational support was diffused by other activities occurring at the community health center during the study period, including simultaneous participation in other quality initiatives, the opening of a new clinic, and major construction projects and upgrades across the organization. Although the director of quality improvement did demonstrate engagement, influence, and capacity, she did not see patients herself and as an RN would have been unable to prescribe statins [lack of credibility]. The three other study implementers did not have clinical backgrounds and lacked the influence, credibility, and capacity (particularly a deep understanding of diabetes and cardiovascular care) to persuade providers to change their behavior; the additional clinician implementer, a practicing physician, did not play an active role in supporting implementation activities.

#### Community health center #3

Implementation efforts at this community health center, a single clinic, were enacted by an RN diabetes care manager who was assigned to the study implementer position. She conducted a few initial presentations about the CVD bundle at provider meetings and occasionally discussed the study’s EHR tools with individual providers. However, as this RN personally met with most of the clinic’s patients with diabetes in her role as a diabetes care manager, most of her implementation activities focused on her own activities. She integrated review of statin prescriptions into her pre-visit chart review process and follow-up visits with diabetic patients, and followed up with the primary care provider to discuss a plan of care based on clinical recommendations. The effectiveness of this approach was limited because (i) the EHR tools were designed to fire during open encounters, not during pre-visit chart reviews; and (ii) the approach relied almost entirely on a single person without prescribing privileges [lack of credibility], with little effort put into raising awareness or buy-in from providers. In addition, although this study implementer actively worked to identify patients with diabetes who could benefit from a statin [engagement] and had the influence and capacity to advocate for the intervention, she occasionally expressed distrust in the guidelines underlying the CVD bundle and/or the algorithm behind the EHR tools. Finally, although this clinic had a standardized process for piloting and approving new interventions and workflows, this process was not applied to the clinic’s participation in SPREAD-NET—which appeared to limit organizational awareness of and support for implementation activities.

## Discussion

Our study adds to implementation science by providing insight into the pathways through which champions may impact implementation outcomes, and advances understanding of how to identify and prepare implementers to be effective champions within their own particular environments. Notably, unlike the community health centers that did not demonstrate a significant change in prescribing behavior, the five community health centers that did have improved outcomes all had engaged and respected champions (study implementers) who were able to directly influence provider behaviors or alter institutional prescribing norms. This suggests that not only the selection of champions as an implementation strategy but also the appropriate operationalization of support (i.e., the identification and preparation of champions), are necessary for effective practice change; this is likely to also apply to other implementation strategies, and further research is needed to identify best practices for doing so. These findings also align with those of other studies that showed the potential for champions to successfully support introducing and maintaining practice change [30–33, 49–52].

Implementation science emphasizes that identifying effective implementation strategies involves understanding the context-specific causal pathways through which these strategies can have impact [3, 34]. The case studies presented here underscore this: effective champions were key to implementation success, but individual differences between study implementers and contextual differences between organizations produced different pathways to change. In some cases, change was effected through hierarchical directives and practice standardization; in others, the champion relied on trust-based relationships, advocacy, and leading by example. This underscores the importance of assessing and reporting how implementation strategies operate in a given setting.

These findings also help explain the overall study results (higher-intensity implementation support was not associated with better outcomes). A recent review [3] found that variation in impact across implementation studies is often due to misalignment between implementation strategies and key contextual barriers and facilitators. A similar phenomenon occurred here: the selected strategy (champions) was appropriate, but as operationalized it had little impact. It was recommended that study clinics appoint study implementers with enthusiasm, credibility, influence, and clinical knowledge, but—in an effort to allow for organizational autonomy—these elements were not required. In addition, the study’s implementation support focused on adoption of the targeted innovation, rather than on increasing the study implementers’ effectiveness. Had the focus been on supporting the development of the implementers’ leadership skills and engagement with the intervention and/or had it been required that study implementers fulfill certain criteria, study results may have been different.

Recognizing this led us to question the process through which we initially selected and operationalized the implementation strategies compared in the SPREAD-NET study. After a period of reflection [53] and extended team discussions, we recognized that a given community health center’s decision to participate in SPREAD-NET had triggered collective, unacknowledged assumptions by our study team regarding organizational support for the targeted change, and study implementer engagement in/capacity to effect that change. We assumed that the implementers designated by the community health centers would have the necessary attributes and qualifications to be effective champions, so the implementation support focused on the specifics of the innovation, and change management strategies. However, these assumptions did not hold true in all study sites, which appears to have influenced study outcomes.

### Implications for implementation science

Social scientists have long argued that articulating tacit assumptions—beliefs that “you accept as true without question or proof” [54]—is necessary to understand the impact of such assumptions on research processes and results [53, 55, 56]. However, consideration of the impact of assumptions on study design and outcomes is largely absent in the implementation of science literature. We contend that reflexivity, or the active querying of one’s own assumptions and related decisions during the design phase—particularly as these assumptions relate to likely barriers and facilitators to intervention uptake—is essential to avoid a mismatch between implementation support and determinants [3].

In addition, despite strong evidence on the importance of champions in implementation activities, little direction exists on how best to support/develop/prepare champions. Often, the literature implies that effective champions have certain intrinsic qualities that cannot be taught [30, 31, 37]. The few articles that do address increasing champions’ efficacy recommend fairly vague strategies such as creating and sustaining learning communities, ongoing mentoring and feedback, fostering the development of leadership and change management skills, and valuing and rewarding champions for their contribution, coupled with hands-on practice and content-specific training [36, 38]. Relevant recommendations note only the need to “identify and prepare individuals who dedicate themselves to supporting, marketing, and driving through an implementation” [27]. A recent article on the attributes of effective champions suggested that many of the necessary skills can be learned, and that supporting the development of these skills may be key to successful implementation outcomes [49].

Our findings also indicate that implementation science theories and frameworks that involve the use of champions should be refined to include detailed specifications on both necessary intrinsic champion attributes and guidance on developing and supporting effective champions. The analysis presented here contributes to the growing knowledge base within the field regarding what makes an effective champion [31, 49, 57]; more research is needed to identify essential champion characteristics, distinguish between those that are context-dependent (e.g., status within the clinic hierarchy) versus those that can be taught, and identify specific, pragmatic techniques that effectively foster necessary skills—all while accounting for the impact of contextual factors on implementation approaches and outcomes.

### Limitations

All of the study community health centers volunteered to participate, and may have shared unique motivations that limit the generalizability of study findings. Qualitative data collection did not occur evenly across community health centers due to lack of engagement and staff turnover at some community health centers, as well as the mid-study closure of one organization. Findings are presented at the organizational rather than clinic level; it is possible that a single clinic within a community health center may have driven the change in cardioprotective prescribing, although we believe this is unlikely. A major finding of the original analysis was that aspects of the CVD bundle itself proved a barrier to implementation [28]. It is possible that weaknesses in the tools yielded a situation in which only sites with strong study implementers were able to make significant progress; better tools might have necessitated less reliance on champions. In addition, while our study used a cluster-randomized design to minimize bias introduced by unrecognized confounders, our randomization scheme was based on available and readily quantifiable factors such as clinic size, urban/rural location, and the prevalence of diabetes. Randomizing by such factors did not, however, ensure equal distribution of the factors ultimately recognized to be associated with differences in study outcomes.

## Conclusion

This analysis adds to implementation science’s call for better approaches to selecting and operationalizing implementation strategies suitable to a given context [2, 5, 9, 11, 15, 26]. Here, unexamined researcher assumptions, coupled with a lack of specification [34] regarding how to prepare effective champions, led to implementation support that failed to address key barriers to success. These results also increase our understanding of the causal mechanisms through which champions may influence implementation outcomes. Implementation practitioners require detailed, pragmatic, context-specific, evidence-based recommendations on how to select and execute implementation strategies; thus, future research should focus on generating evidence on how to support the growth of effective champions.

## Supplementary information

**Additional file 1.** SRQR guidelines for reporting qualitative research studies.

## Data Availability

The quantitative data analyzed during the current study will not be made publicly available but are available from the corresponding author on reasonable request. The qualitative data analyzed during the current study are not publicly available due to them containing information that could compromise research participant privacy; the codebook and data collection tools are available on request.

## Abbreviations

CVD: Cardiovascular disease
DM: Diabetes mellitus
EHR: Electronic health record
